# Severe Hemolytic Anemia due to Vitamin B12 Deficiency in Six Months

**DOI:** 10.3390/hematolrep14030028

**Published:** 2022-06-22

**Authors:** Narayanan Sadagopan

**Affiliations:** University of Massachusetts Medical School, Worcester, MA 01655, USA; narayanan.sadagopan@umassmemorial.org

**Keywords:** vitamin B12, hemolysis, anemia

## Abstract

Gastric bypass is a common cause of vitamin B12 deficiency. It can lead to patients presenting with symptoms of anemia. The body has significant reserves of vitamin B12 and loses vitamin B12 slowly. The following case is of a patient who underwent a gastric bypass five years ago and whose hemoglobin (Hgb) dropped from 12.2 g/dL to 4.4 g/dL over six months due to questionable adherence to vitamin supplements. Further work-up showed hemolytic anemia and thrombocytopenia due to a very low vitamin B12 level of 47 pg/mL, with his blood counts improving with vitamin B12 supplementation. The case points to the importance of thinking about vitamin deficiency as a cause of hemolysis to avoid unnecessary procedures.

## 1. Introduction

Vitamin B12 deficiency is a common cause of macrocytic anemia. It is often seen in patients following bariatric surgery which is why vitamin B12 supplementation is recommended for these patients [1]. It is estimated that about 0.13% of the body’s total vitamin B12 store is lost per day [2]. Consequently, it takes a while to deplete the body’s vitamin B12 store, even with zero vitamin B12 intake. Vitamin B12 deficiency can lead to ineffective erythropoiesis and intramedullary hemolysis. In one cohort of patients with vitamin B12 deficiency, it led to severe anemia defined as Hgb < 6 g/dL in 2.5% of the patients and hemolytic anemia in 1.5% of the patients [3]. This presentation can mimic a thrombotic microangiopathy [4]. Quickly being able to identify vitamin B12 deficiency as the cause can lead to simple therapy with vitamin B12 replacement and avoid unnecessary treatments such as plasma exchange. The following is a case of potential life-threatening hemolysis due to vitamin B12 deficiency that developed relatively quickly and resolved with vitamin B12 supplementation.

## 2. Case

The patient is a 52-year-old man with a past medical history of gastric bypass 5 years ago who presented at the emergency room with worsening shortness of breath and fatigue for about 1 month. He was previously able to walk upstairs without issue, but when presenting with these symptoms almost exclusively had to the use the elevator. His previous labs were notable for Hgb 12.2 g/dL six months prior to presentation. He stated he was supposed to be taking vitamin supplements after the gastric bypass but has mostly been non-adherent. Labs on presentation were notable for a Hgb 4.4 g/dL (normal 13.2–17) with hematocrit (HCT) 12.7% (normal 38–48). He was transfused 3 units of packed red blood cells with improvement in his Hgb to 6.7 g/dL. Other lab findings indicated hemolysis with a total bilirubin of 2.4 mg/dL (normal 0.3–1.2), indirect bilirubin 2.1 mg/dL (normal 0.2–0.8), lactate dehydrogenase (LDH) 2190 U/L (normal 110–240), and haptoglobin < 8 mg/dL (normal 43–212). He was Coombs negative and reticulocyte index on presentation was 0.21. Peripheral blood smear was just notable for hyper segmented neutrophils without schistocytes. His mean cell volume (MCV) was 128.3 fL, pointing to potential vitamin B12 or folate deficiencies. Iron studies were notable for ferritin 233 ng/mL (normal 26–388), iron 182 μg/dL (normal 65–175), iron binding capacity 186 μg/dL (normal 250–450), and %saturation of 98 (normal 20–55). Folate was 17.33 ng/mL (normal > 5.4) and his vitamin B12 was found to be very low at 47 pg/mL (normal 211–911). He also had moderate thrombocytopenia in the 70–110 10^3^/μL range. He received treatment with intramuscular vitamin B12 1000 mcg daily for 3 days. His energy level started to improve and on discharge from the hospital on day three his lab tests showed Hgb 7.3 g/dL, HCT 20.6%, platelets 77 10^3^/μL, MCV 101 fL, indirect bilirubin 1.6 mg/dL, and LDH 1849 U/L. He wanted oral supplementation only, so he was discharged on 2 g of vitamin B12 daily. Labs 5 days later showed Hgb 8.3 g/dL, HCT 25.2%, platelets 153 10^3^/μL, and MCV 102.4 fL. He was continued on the 2 g daily of vitamin B12. Then, 2 months later, Hgb 12.9 g/dL, HCT 39.6%, platelets 177 10^3^/μL, MCV 88.7 fL, and vitamin B12 level > 2000 pg/mL, so his daily B12 was reduced to 100 mcg. He never had any paresthesia’s or gait disturbances. His shortness of breath and fatigue had completely resolved by this point.

## 3. Discussion

The patient’s Hgb drop from 12.2 g/dL to 4.4 g/dL over 6 months just due to vitamin B12 deficiency is quite surprising. Such a considerable drop over that short of a period is unexpected. This does coincide well with his presenting typical anemia symptoms of progressive shortness of breath and fatigue. He is at higher risk for B12 deficiency due to his prior gastric bypass procedure. Although he had no history of Hgb below 12 g/dL beforehand, he likely had very little vitamin B12 intake during those 6 months for his Hgb to drop as it did. It is possible he went from occasionally taking vitamin B12 supplements at home to not taking any at all over the past 6 months, but he did not give a clear answer. Since it usually takes 2–5 years to exhaust liver and kidney reserves of vitamin B12 [5,6], that timeline aligns with his gastric bypass 5 years ago, leading to his reserves being depleted.

Vitamin B12 is a cofactor for methionine synthase which catalyzes the conversion of homocysteine to methionine and 5-methyl tetrahydrofolate to tetrahydrofolate [7]. Tetrahydrofolate is crucial for DNA synthesis. Vitamin B12 deficiency leads to impaired nuclear division while cytoplasmic maturation is less impaired leading to the classical megaloblastic anemia [8]. Hemolysis in vitamin B12 deficiency is thought to be related to elevated levels of homocysteine which build up as homocysteine cannot be converted to methionine. In vitro homocysteine was found to cause hemolysis by possible lipid peroxidation and cytoskeletal protein derangement [9]. Vitamin B12 deficiency can also lead to neurologic effects with peripheral neuropathy and degeneration of the corticospinal tracts and posterior columns [10]. His lack of neurologic symptoms may point to prolonged vitamin B12 deficiency being more related to neurologic symptoms instead of the severity of the vitamin B12 deficiency.

His presentation of worsening fatigue and worsening shortness of breath coincides with a steadily declining Hgb level. A sudden decrease in Hgb such as his typically prompts considering other conditions in the initial differential such as acute blood loss or hematologic malignancy. In addition, given his initial labs were indicative of hemolysis and thrombocytopenia, life-threatening conditions such thrombotic thrombocytopenic purpura must be considered. His MCV of 128.3 fL on the initial complete blood count was the main factor that pointed to a potential vitamin deficiency. This allowed prompt identification of his very low vitamin B12 levels and treatment with vitamin B12, resulting in improvement in his Hgb. This also prevented unnecessary procedures such plasma exchange, colonoscopy, or bone marrow biopsy. His peripheral blood smear with hyper segmented neutrophils and without schistocytes also pointed away from some type of thrombotic microangiopathy. Schistocytes are quite specific for the diagnosis of a thrombotic microangiopathy, as erythrocytes become damaged when traveling through vasculature obstructed by microthrombi [11]. Though rarely there can be cases of conditions such as thrombotic thrombocytopenic purpura without schistocytes [12], he likely would have had a more profound thrombocytopenia given his presenting hemoglobin if it was all due to a thrombotic microangiopathy. His case may point towards elevated homocysteine levels from his vitamin B12 deficiency causing the hemolysis, instead of vasculature obstructed by microthrombi. Nonetheless, some schistocytes would have been expected given his presenting labs indicating hemolysis. His Hgb returning to baseline with just vitamin B12 supplementations points to no other underlying cause of his anemia other than the vitamin B12 deficiency. Hemolysis markers such as LDH and indirect bilirubin both started to decrease after starting vitamin B12 supplementation. In conclusion, vitamin B12 deficiency should be considered as a cause of even severe hemolysis, especially in patients with pre-existing risk factors for B12 deficiency.
